# Supplementary figures and images for: Structures of immature EIAV Gag lattices reveal a conserved role for IP6 in lentivirus assembly
Source: PLoS Pathog. 2020 Jan 27;16(1):e1008277. doi: 10.1371/journal.ppat.1008277 (PMC7004409; doi:10.1371/journal.ppat.1008277)

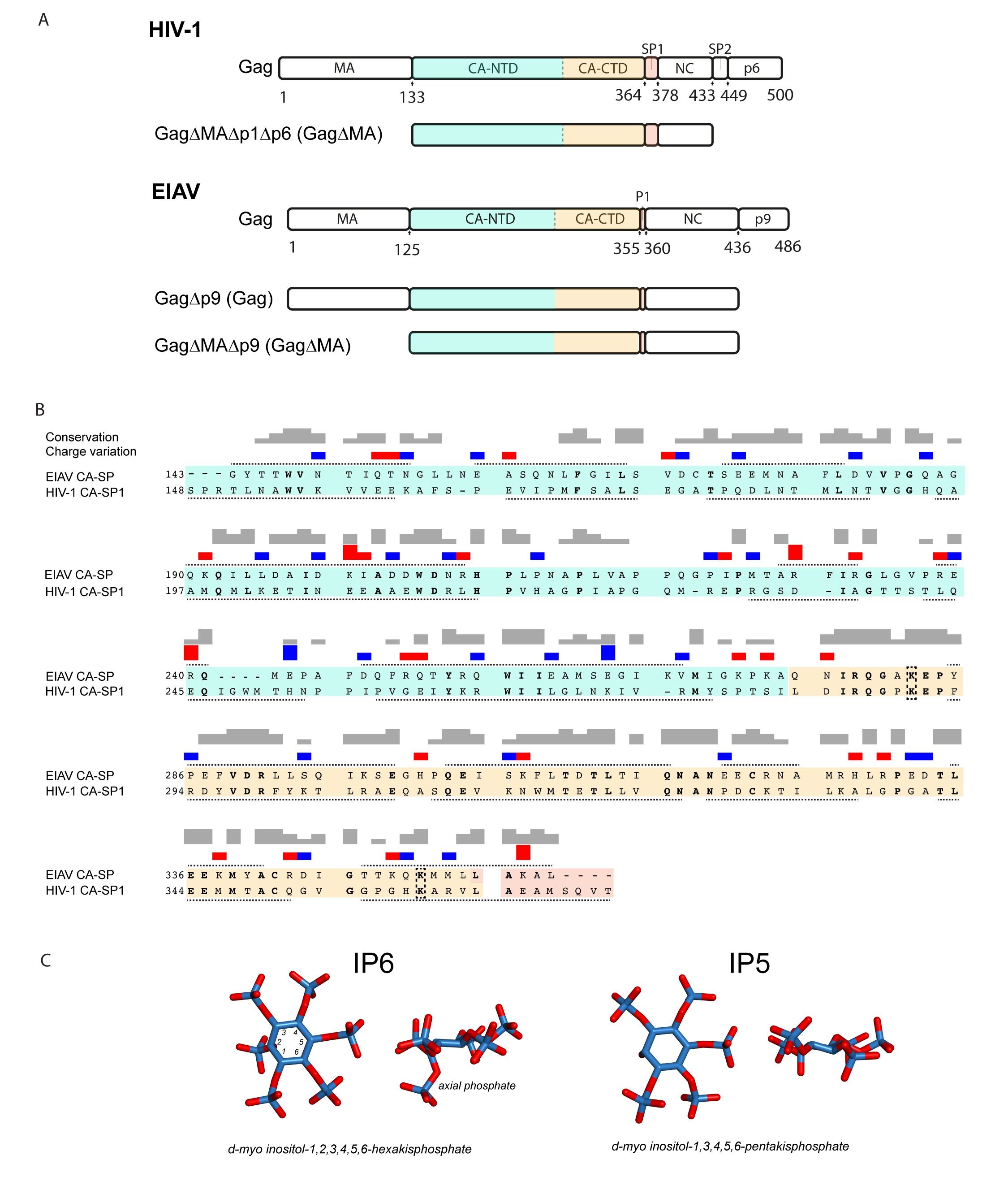

Supplement: S1 Fig — (A) Schematic representation of HIV-1 and EIAV Gag, and the truncated Gag variants used in this study. The cleavage sites are annotated by arrows and the first residue of each individual domain is shown. The CANTD and CACTD are colored cyan and orange, respectively. The SP region is colored red. Abbreviated protein names in parenthesis. (B) Sequence alignment between HIV-1 CA-SP1 and EIAV CA-SP. The background of the sequence is colored as in (A) to indicate the location of the CANTD, CACTD or SP. Secondary structure assignments (alpha-helices) are indicated with dashed lines. Above the sequence alignment the conservation of the respective residues and existing charge variations are shown, in gray and blue/red respectively; a blue bar indicates a positive charge variation from EIAV to HIV-1, conversely a red bar indicates a negative charge variation from EIAV to HIV-1. Conserved lysine residues are in indicated by dashed rectangles. (C) Molecular representations using the licorice representation of IP6 and IP5. It is clearly shown that IP5 lacks the axial phosphate present in IP6. (TIF) [file ppat.1008277.s001.tif]

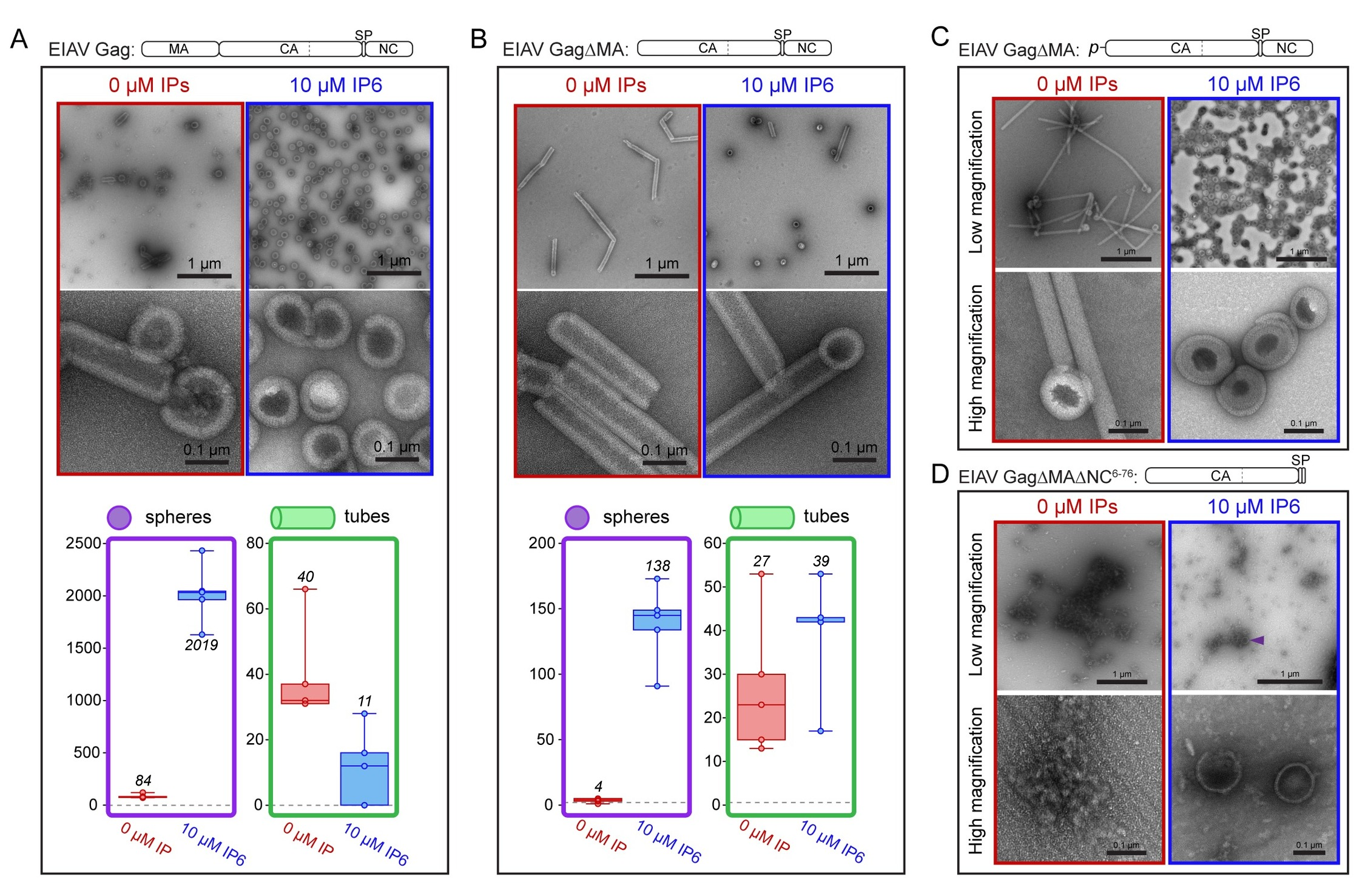

Supplement: S2 Fig — (A,B) Representative low and high magnification images of Gag and GagΔMA assembled in the absence (red) or presence (blue) of 10 μM IP6 at pH 8. The number of VLPs (spheres-purple, tubes-green) per 55μm2 for no fewer than five representative images for each condition. Center lines show the medians; box limits indicate the 25th and 75th percentiles as determined by R software; whiskers extend to minimum and maximum values; data points are plotted as circles. (C) Representative low and high magnification images of native GagΔMA assembled in the absence (red) and presence (blue) of 10 μM IP6 at pH 6. Tubes assembled in the absence of IP6 were the same, by negative stain EM, as tubes assembled with GagΔMAΔp9 containing an ectopic serine at the N-terminus. In the presence of IP6, protein formed multi-layered, spherical VLPs. (D) Representative low and high magnification images of GagΔMAΔNC6-76 assembled in the absence (red) or presence (blue) of IP6 at pH 6. Very few VLPs were observed; see purple triangle, compared to the amount observed for HIV-1 [17]. The mean value of counted particles is given in italics in the bar charts. (TIF) [file ppat.1008277.s002.tif]

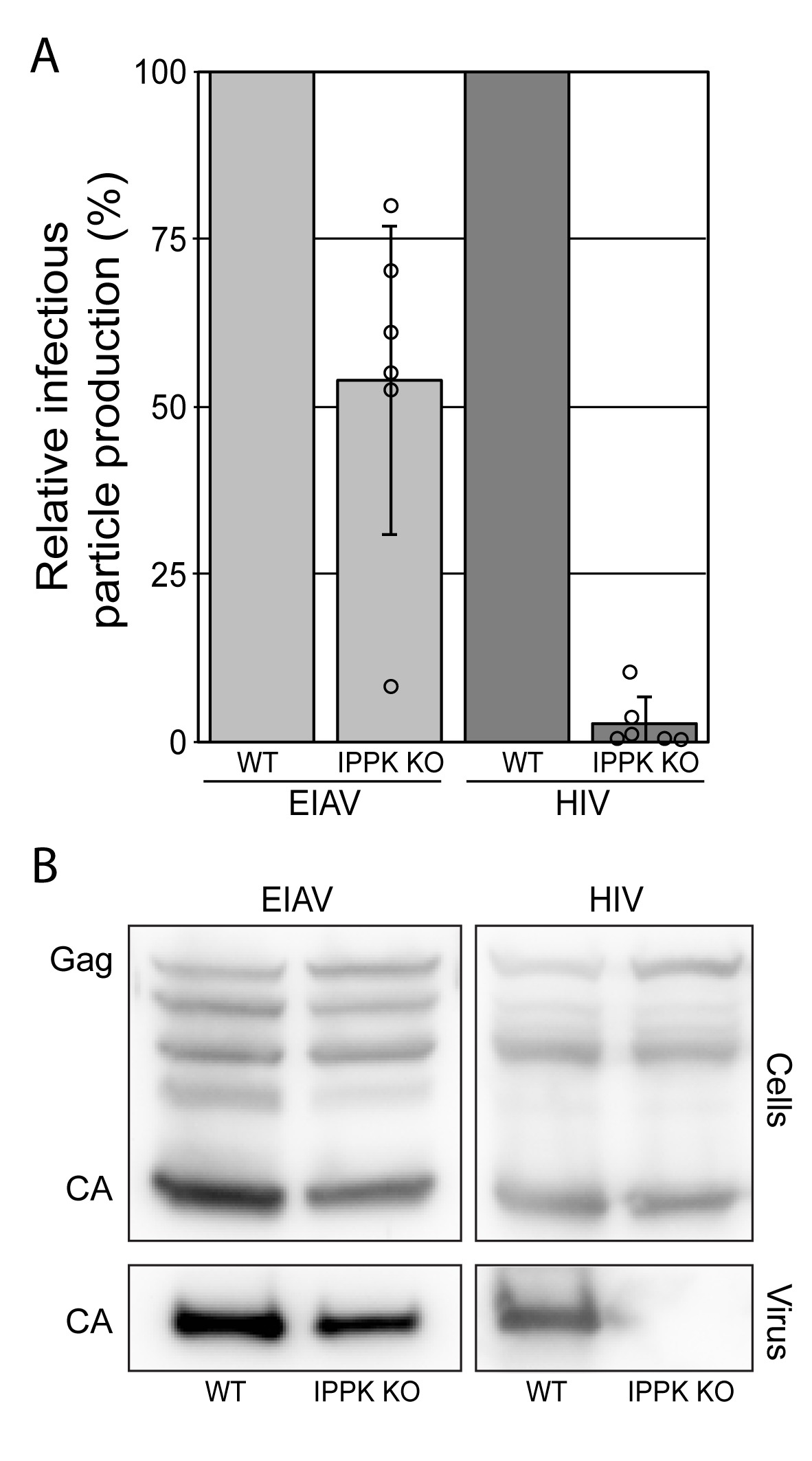

Supplement: S3 Fig — (A) The effect of IPPK KO on the production of infectious EIAV and HIV virus particles. Relative to WT, bars represent the average and whiskers the standard deviation of no fewer than three replicates. (B) Western blots were performed on cell lysates and released virus from HEK293T WT and IPPK KO cells for EIAV and HIV. EIAV was probed with RbαEIAV-CANC and HIV with MsαHIVp24. (TIF) [file ppat.1008277.s003.tif]

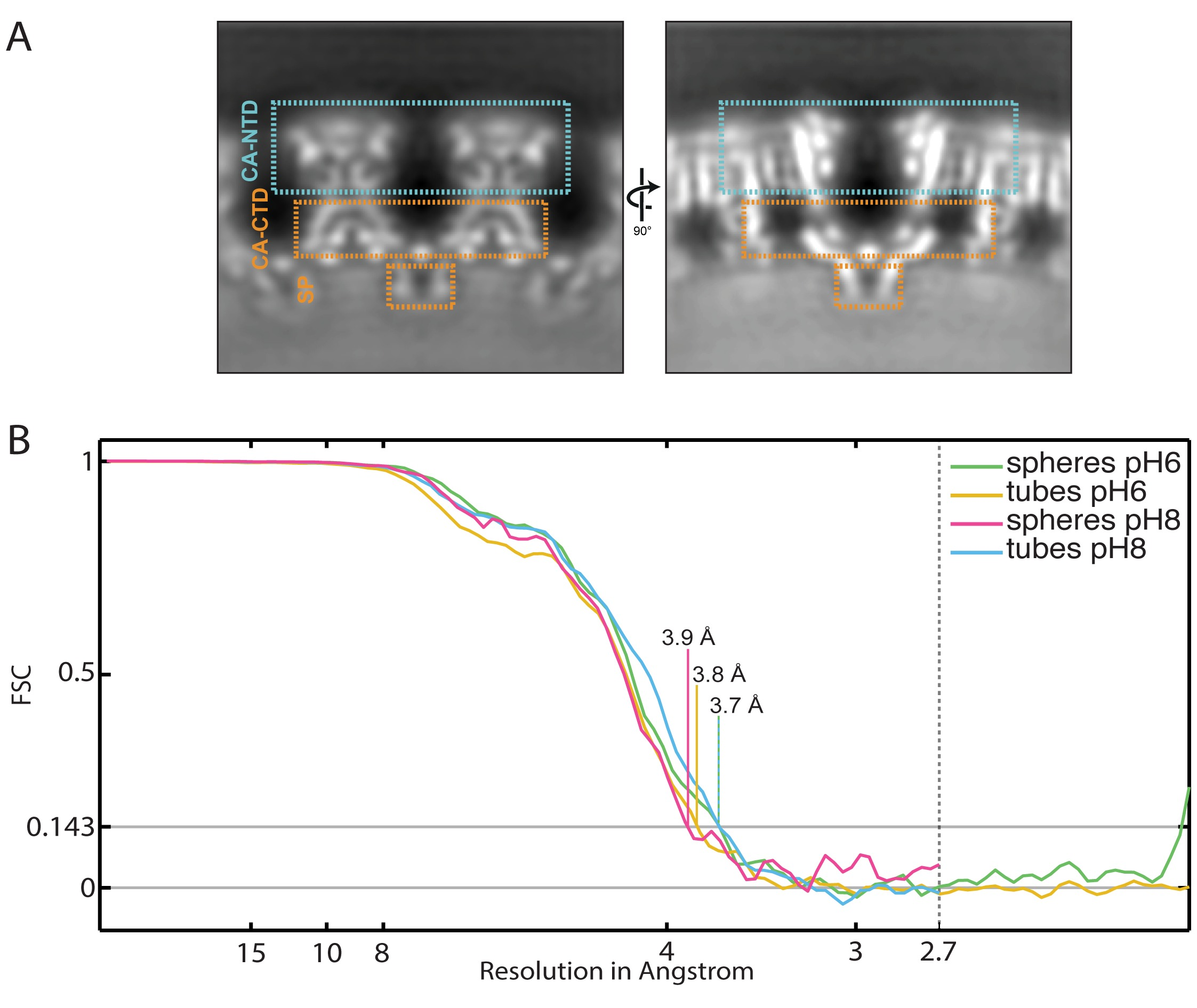

Supplement: S4 Fig — (A) Radial orthoslices through the final sharpened map from spheres assembled at pH6, filtered to 8 Å resolution. Protein density is white. The level of the CANTD, CACTD and SP is indicated with dashed boxes. (B) Fourier shell correlations (FSC) between independent halfsets for EIAV GagΔMA spheres (green) and tubes (orange) assembled at pH6, and spheres (pink) and tubes (blue) assembled at pH8. The resolution measured at the 0.143 FSC criterion is indicated with lines. The Nyquist frequency for the dataset of particles assembled at pH8, which was acquired with a pixel size of 1.35 Å is indicated at 2.7 Å. (TIF) [file ppat.1008277.s004.tif]

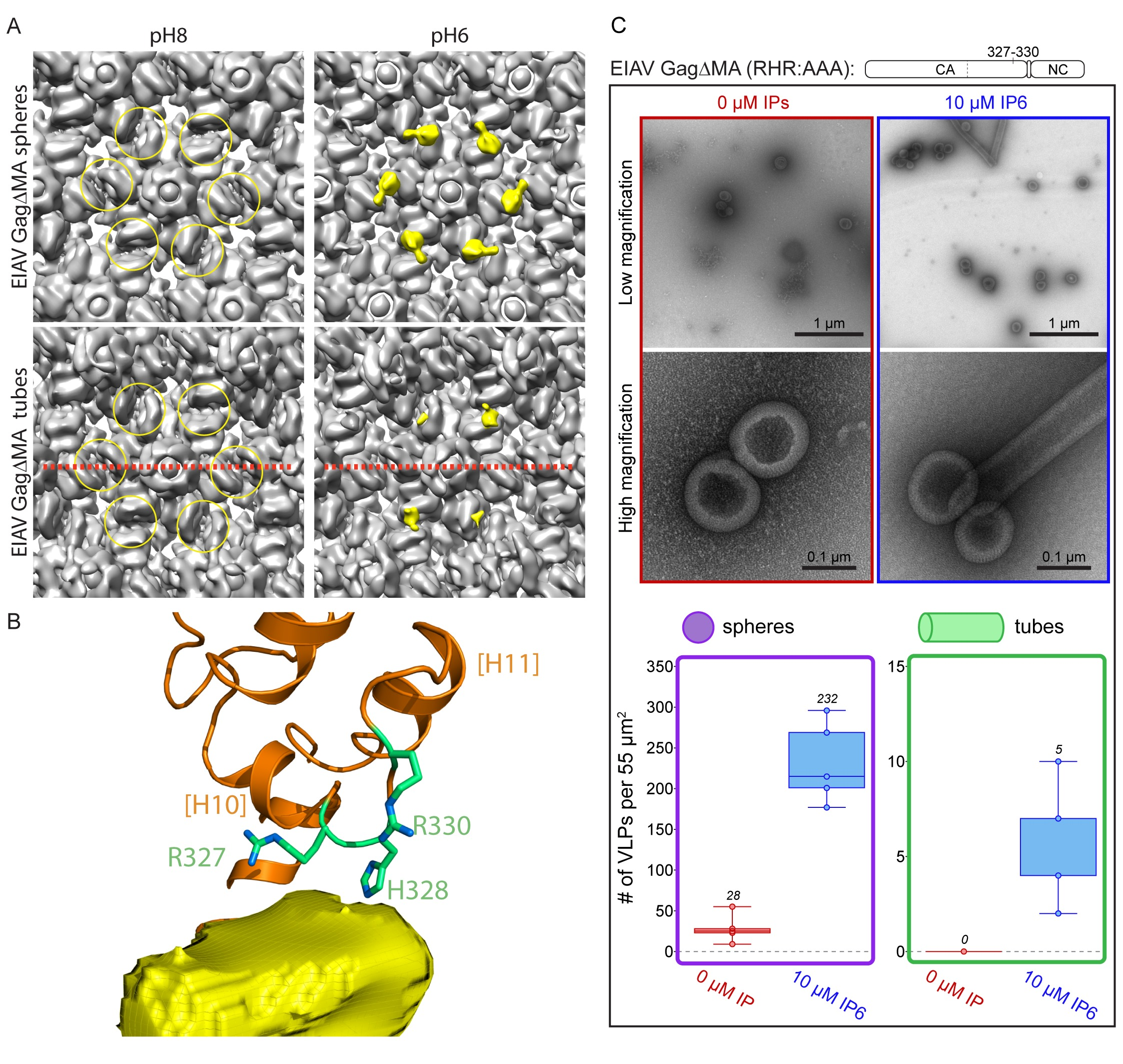

Supplement: S5 Fig — A) Isosurface representations of EIAV CA-SP from spherical and tubular assemblies at pH8 and pH6 as seen from the inside of the particle. All structures have been filtered to 8 Angstrom resolution. The additional densities in the spheres and tubes assembled at pH6 are highlighted in yellow. The corresponding positions in the sphere structure assembled at pH8 are circled in yellow. The red dashed line indicates the tube axis, showing that the additional densities are absent along the direction of the tube, where no curvature between the CACTD dimer is found. This observation of an additional density being recruited to the base of the CACTD by basic residues is reminiscent of a similar interface observed in M-PMV [75]. B) The three positively charged residues (green) in the linker connecting helix 10 and helix 11 are positioned to interact with the additional density (displayed as an isosurface in yellow). C) (Top) Low and high magnification negative staining TEM images of GagΔMA (RHR:AAA) VLPs assembled at pH 6 without (red) and with (blue) 10 μM IP6. (Bottom) Quantification of the number of VLPs counted for no fewer than five representative TEM images at pH 6 without and with 10 μM IP6. The mean value of counted particles is given in italics in the bar charts. (TIF) [file ppat.1008277.s005.tif]

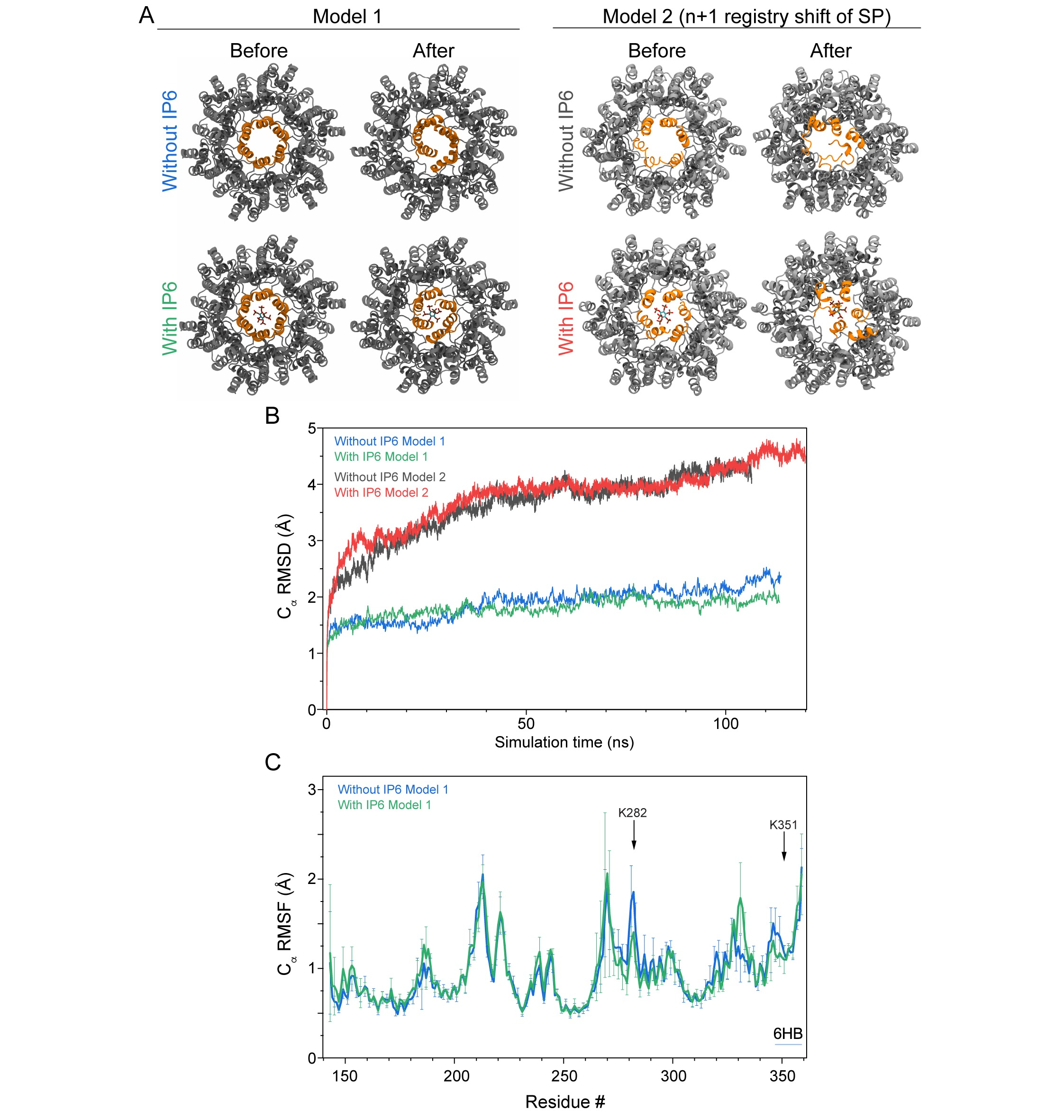

Supplement: S6 Fig — A) Structural changes observed following ~150 ns of MD simulations of CACTDSP without and with bound IP6 for model 1 (correct model) and model 2 (model with registry shift in the SP helix). We ran one simulation with an incorrect model with a n+1 registry shift of the SP helix, clearly showing the loss of 6HB bundle stability and partial unfolding. B) Root mean squared deviations (RMSDs) of the central hexamer during simulations. C) Root mean squared fluctuations were averaged over six central monomers for model 1 with standard deviations shown for each residue. RMSFs are good indicators of protein flexibility. (TIF) [file ppat.1008277.s006.tif]

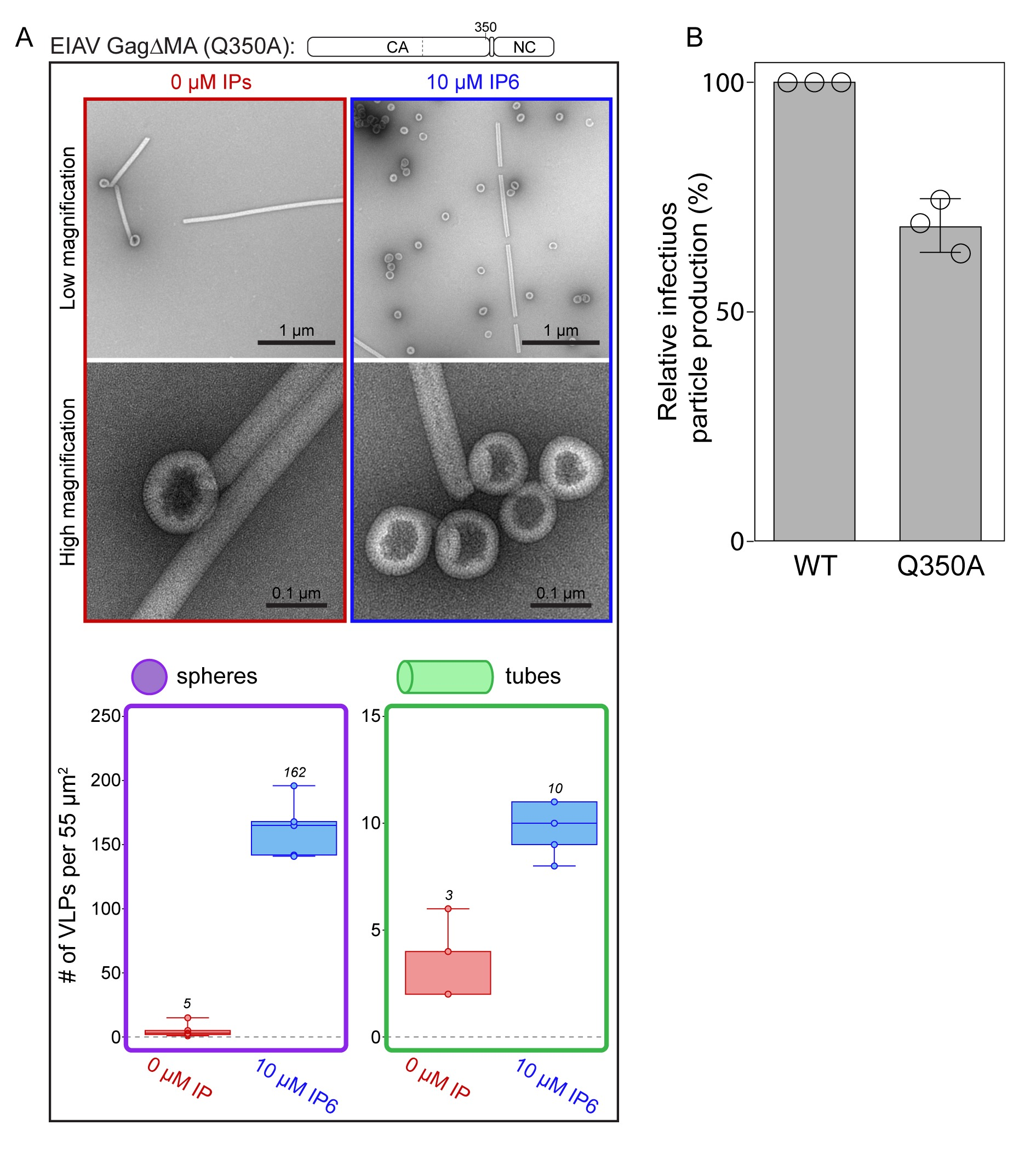

Supplement: S7 Fig — (A) Representative low and high magnification negative stain EM images of in vitro assembled GagΔMA (Q350A) in the absence (red) and presence (blue) of 10 μM IP6. Compared to GagΔMA (Fig 1), the number of VLPs formed in the absence and presence of IP6 is much lower. However, the stimulatory effect of IP6 is still apparent. (B) Relative infectious particle production of wild type EIAV Gag (WT) and Gag (Q350A) point mutation VSV-G-pseudotyped provirus in 293FT cells. Graphs show the average and standard deviation of three independent experiments; dots show individual data points. The mean value of counted particles is given in italics in the bar charts. (TIF) [file ppat.1008277.s007.tif]

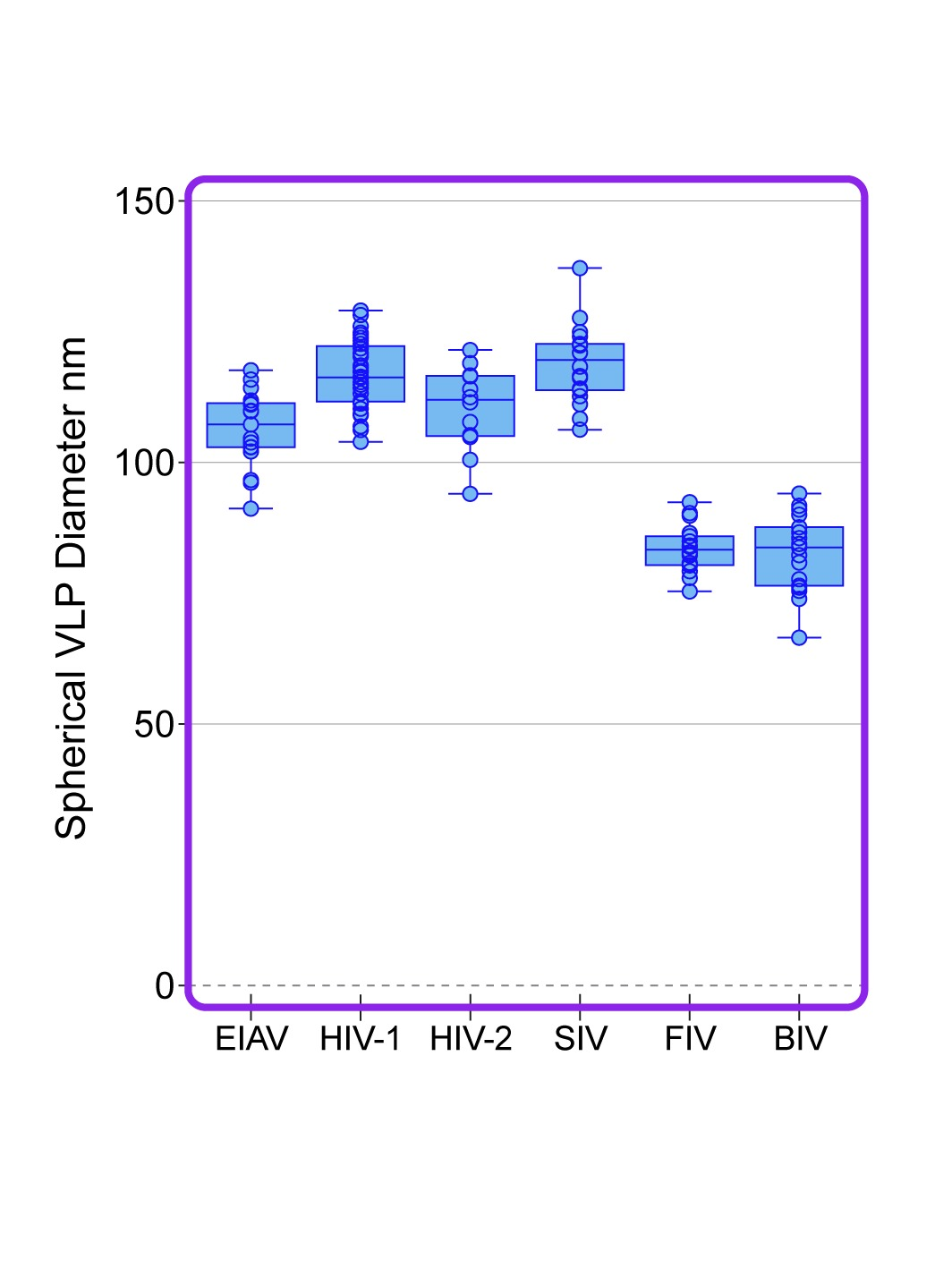

Supplement: S8 Fig — In vitro assembled VLPs were imaged via negative stain EM. From the latter the diameters of the particles were determined. (TIF) [file ppat.1008277.s008.tif]
