# Supplementary material for: Structures of immature EIAV Gag lattices reveal a conserved role for IP6 in lentivirus assembly
Source: PLoS Pathog. 2020 Jan 27;16(1):e1008277. doi: 10.1371/journal.ppat.1008277 (PMC7004409; doi:10.1371/journal.ppat.1008277)
Supplement: S1 Table — (PDF) [file ppat.1008277.s009.pdf]

Sample		EIAV Gag∆MA spheres pH6	EIAV Gag∆MA tubes ph6	EIAV Gag∆MA spheres ph8	EIAV Gag∆MA tubes ph8
**Acquisition settings**	**Microscope**	FEI Titan Krios	FEI Titan Krios	FEI Titan Krios	FEI Titan Krios
	**Voltage (keV)**	300	300	300	300
	**Detector**	Gatan Quantum K2	Gatan Quantum K2	Gatan Quantum K2	Gatan Quantum K2
	**Energy-filter**	Yes	Yes	Yes	Yes
	**Slit width (eV)**	20	20	20	20
	**Super-resolution mode**	Yes	Yes	Yes	Yes
	**Å/pixel**	1.041	1.041	1.35	1.35
	**Defocus range (microns)**	-1.5 to -3.5	-1.5 to -3.5	-1.5 to -5.0	-1.5 to -5.0
	**Defocus step (microns)**	0.25	0.25	0.25	0.25
	**Acquisition scheme**	-60/60°, 3°, Serial EM	-60/60°, 3°, Serial EM	-60/60°, 3°, Serial EM	-60/60°, 3°, Serial EM
	**Total Dose (electrons/Å^2)**	~140	~140	~140	~140
	**Dose rate (electrons/Å^2/sec)**	~2.5	~2.5	~2.5	~2.5
	**Frame number**	21	21	20	20
	**Tomogram number**	40	20	55	56
**Processing settings**	**VLPs**	175	152	158	106
	**Asymmetric units**	389,766	225,858	394,842	179,720
	**B-factor used for sharpening**	-270	-150	-245	-150
	**Final resolution (0.143 FSC) in Å**	**3.7**	**3.8**	**3.9**	**3.7**
