# Supplementary material for: Structures of immature EIAV Gag lattices reveal a conserved role for IP6 in lentivirus assembly
Source: PLoS Pathog. 2020 Jan 27;16(1):e1008277. doi: 10.1371/journal.ppat.1008277 (PMC7004409; doi:10.1371/journal.ppat.1008277)
Supplement: S2 Table — (PDF) [file ppat.1008277.s010.pdf]

Refinement	EIAV GagΔMA spheres pH6 residues 143-359	EIAV GagΔMA tubes ph6 residues 143-359	EIAV GagΔMA tubes ph8 residues 143-359
**Resolution of map (Å)**	3.7	3.8	3.7
**Number of CA-SP monomers**	1	3	3
**All-atom clash score**	6.78	2.91	5.91
**Favored rotamers**	93.58%	91.27%	89.30%
**Ramachandran outliers (number of residues)**	0	0.31% (2)	0
**Ramachandran favoured**	93.95%	92.71%	91.94%
**C-beta deviations**	0	0	0
**Rmsd (angles, degrees)**	0.861	0.839	0.917
**Rmsd (bonds, Å)**	0.006	0.005	0.006
