# Supplementary material for: Structures of immature EIAV Gag lattices reveal a conserved role for IP6 in lentivirus assembly
Source: PLoS Pathog. 2020 Jan 27;16(1):e1008277. doi: 10.1371/journal.ppat.1008277 (PMC7004409; doi:10.1371/journal.ppat.1008277)
Supplement: Attachment — Submitted filename: Response_to_Reviewers.docx [file ppat.1008277.s015.docx]

We thank the reviewers for their valuable comments and the editors for allowing us to revise our manuscript. As requested, we have undertaken the necessary corrections and also performed the required experiments to quantify EIAV and HIV-1 Gag levels in cells and virus fractions. We have, due to the experimental efforts and in agreement with editorial guidance, refrained from repeating cryo-electron tomography and subtomogram averaging experiments of EIAV Gag-deltaMA assemblies in the absence of IP6.

**Part II – Major Issues: Key Experiments Required for Acceptance**
Please use this section to detail the key new experiments or modifications of existing experiments that should be absolutely required to validate study conclusions.

Generally, there should be no more than 3 such required experiments or major modifications for a "Major Revision" recommendation. If more than 3 experiments are necessary to validate the study conclusions, then you are encouraged to recommend "Reject".


Reviewer #1: 1. Supp Fig. 3. The authors measure the effect of IPPK KO on EIAV and HIV-1 particle production by measuring the infectivity of virus produced from KO cells. As mentioned above, this approach does not differentiate between effects on particle production and effects on infectivity. The authors need to perform virus assembly assays in which levels of HIV-1 and EIAV Gag in the cell and virus fractions are quantified so that virus release efficiency can be determined, a infectivity values need to be normalized based on viral input (measured by RT or capsid protein).

We thank the reviewer for this suggestion. We have in the meantime obtained an anti-EIAV CANC antibody, which allowed us to measure EIAV Gag levels in cells and virus fractions, in order to determine the efficiency of virus release.

Consistent with our *in vitro* observations, for EIAV we see no large difference in the amount of virus released from FT or IPPK KO cells via western blot analysis (now added to supplemental figure 3), while for HIV we see a dramatic decrease in the amount of virus released from FT compared with IPPK KO cells. Thus, we can assign the bulk of the effect on decreased infectivity in IPPK KO cells to a defect in virus release.

We have modified the manuscript to reflect these points in the following ways:

a) A panel has been added to supplemental figure 3 showing the results of the western blot assays for both EIAV and HIV. Representative western blots are shown, comparing released virus with intracellular Gag.

b) The results section has been modified at lines 171-177:

“*Western blots demonstrate that the release of EIAV from cells was not significantly different between control and IPPK KO cells (Supplemental Fig 3B). These results are consistent with the in vitro assembly data showing that that immature EIAV assembly is enhanced by, but not dependent on, IP6. For HIV-1 the western blots show a significant decrease in virus release from IPPK KO cells compared to control cells. We interpret this to mean that the large reduction in infectious virus particle production for HIV-1 is due to a reduction in immature virus particle release.*”

Methods section has been accordingly modified at lines 546-548 and supplemental Figure 3 legend has been modified at lines 905-907.

2. P. 16. The authors speculate about the role of IP6, or potentially other positively charged molecules, in EIAV assembly in vivo. They suggest that, in the cells used, EIAV infectivity is significantly lower than that of HIV-1. Again, this issue needs to be addressed by measuring the efficiency of virus particle production and determining the relative infectivity (normalized for levels of RT or CA) of the EIAV stocks. These crucial experiments will resolve this issue.

Please see the above section in which we describe the western blot results for EIAV.

Consistent with our own assays, it was previously reported that EIAV produces ~100-fold fewer infectious units than HIV (REF-40). The western blots referred to above imply that that similar amounts of EIAV are released from both the FT and IPPK KO cells; however, since the infectivity for WT is already very low, it is likely that much of the released virus from both cell types is non-infectious. We have added REF-40 to line 444 of the text.

3. Line 425. The authors discuss how future work will evaluate the effect of IP6 depletion on assembly, budding and infectivity. This work will need to be performed by measuring virus release efficiency and specific particle infectivity as described above.

We thank the reviewer for this suggestion, and agree. As stated above it appears that for HIV the bulk of the effect of IP6 depletion on infectious virus particle production is at the formation and/or release of the immature virus. This does not mean that IP6 is not required for mature virus particle formation or infectivity. To address this question, it would be necessary to find an HIV mutant that this not dependent on IP6 for immature assembly and virus release. We are still working to identify an experimental approach to address this question.

4. Supp Fig. 7. Have the authors measured the efficiency of virus particle production of the Q350A mutant in IPPK KO cells? This result would be interesting.

We have not performed this experiment since Q350 is not involved in a direct interaction with IP6. This mutation might display an additive (or, less likely, a compensatory) effect in the IPPK KO cells. However, it would be difficult to disentangle the contribution of each of these variables on particle release, leading only to speculative interpretations.

Reviewer #2: My only major concern is related to the assignment of the density for IP6. In order to be certain that this density corresponds indeed to IP6, the authors should obtain an immature lattice average without it (i.e. a negative control), for example using the EIAV GAGdeltaMA preparation with 0µM IP. This is specially required as IP6 sits at the symmetry axis, and non-relevant densities can appear there due to symmetrisation.

The reviewer is right that densities positioned on symmetry axes can become overemphasized, but we do not believe that has happened in this case. Firstly because because the density is strong, and the symmetry axis is otherwise free of symmetrization artefacts. Secondly, because the shape and size of the density clearly resembles IP6 in its myo-conformation, which we have recently resolved in our HIV-1 CA-SP/IP6 co-crystal structure (Dick RA et al., 2018). Thirdly, because the density is found at exactly the same position and has the same shape and size in spherical particles where six-fold symmetry is applied and in tubular particles where only two-fold symmetry is applied, making it unlikely that it is a higher-order symmetrization artefact. In the revised version of the manuscript we have commented:

*Line 316: ‘The shape and size of the IP6 density is similar in tubular and spherical particles. The better-resolved density in the map from GagΔMA spheres assembled at pH6 has a strikingly similar shape and size to the density observed for IP6 in the recently obtained co-crystal structure of HIV-1 CACTD-SP with IP6.’*

Based on editorial guidance and due to the significant additional experimental efforts that would be required, we have not sought to obtain an immature lattice average without IP6. We do not think the density can reasonably be contributed by another molecule from the well-defined in vitro assembly reaction.

Reviewer #3: Comments, concerns or questions:
1) Although stimulated by IP6, in vitro assembly of EIAV Gag can happen without presence of IP6 (line 331-335). The authors think this is because that in EIAV most of the interactions of the six-helix bundle are hydrophobic in nature, which strengthen the six-helix bundle structure. In contrast to HIV-1, “MD simulations the EIAV 6HB was stable both with and without IP6 likely due to additional side chain contacts between helices in the six-helix bundle” (line 404-405). Can you perform a sequence alignment of this region of all lentiviruses studied in the paper and address if this property is unique for EIAV?

We have extended the alignment of Figure 6. However, this does not allow us to conclude whether the property for EIAV is unique because it depends upon helix register and orientation. In the absence of structural data for the other lentiviruses, we prefer not to speculate further on this point.

**Part III – Minor Issues: Editorial and Data Presentation Modifications**
Please use this section for editorial suggestions as well as relatively minor modifications of existing data that would enhance clarity.


Reviewer #1: Minor: Fig. 6A. The sequence of SP1 is incorrect, as is the placement of the CA-SP1 cleavage site. The correct sequence is KARVL/AEAMSQ, where / denotes the cleavage site.

We have corrected this mistake in the revised version of the manuscript.

Reviewer #2: - Can the authors estimate the occupancy of IP6 in their averages?

This is a valuable suggestion. We have currently no experimental means to reliably determine the occupancy of IP6 in the averages. However, as the strength for the IP6 density is comparable to the CA-SP protein densities surrounding it, one can confidently assume that IP6 binding is taking place in the majority of hexamers that have contributed to our final average. We have added the sentence (line 200-201) “*The density is similar in strength to that of the surrounding protein, suggesting that the majority of hexamers contain a bound molecule.* “

- It would be good if the authors could provide a list/table of the conditions attempted to produce a mature EIAV lattice. Related to this, there is a reference missing in line 473

We have added a description of the conditions we tested in the text (lines 497-501).

Reviewer #3: Comments, concerns or questions:

2) Line 264-269: EIAV CA was found to have a shorter helix 1, and therefore in EIAV Gag lattice, the putative dimer interface between neighboring Gag hexamers (found in HIV-1 lattice) is missing. According to HIV-1 mature CA lattice, helix 1 is important in intra-hexamer interactions. Could this be one of the reasons that in the in vitro assembly systems, EIAV Gag always form immature lattice, not mature lattice? Is this property unique for EIAV?

The putative dimer interface between Helices 1 of EIAV is not missing because it is shorter, but rather because of an overall larger separation of Helices 1, compared with HIV-1 (irrespective of the tubular or spherical EIAV assembly phenotypes). “*Here we show that in EIAV such a dimeric interaction is not involved in determining the immature lattice, as the distance between helices 1 is highly variable between the different assembly morphologies (Fig 3E).”*

Forming a mature EIAV lattice would require folding of the first turns of Helix 1. This is in line with a previous report showing that the initial residues of EIAV CA only become helical upon beta-hairpin formation (reference 32 in our manuscript, Chen K, et al., J Biol Chem. 2013 Jan;288(3):1511–20.). Among these residues is an Arginine, which is conserved among lentiviruses and has been shown to bind IP6 in the mature HIV-1 CA-NTD binding pocket. However, it remains a matter of pure speculation why or if EIAV requires a refolding of Helix 1 upon maturation, and we therefore have not included this in our discussion.

We do not know whether refolding of Helix 1 is unique for EIAV. This question can be answered by obtaining additional structural information on the immature assemblies of the remaining lentiviruses. This represents an exciting research direction for future experiments.

3) The authors found that the EIAV CA-NTD organization is not the same as that in HIV-1. Several molecular contacts in HIV-1 Gag lattice are missing in EIAV which include: (a) there is no intra-hexamer interactions among CA-NTD (line 238-239) (b) there is no dimeric inter-hexamer interaction formed by helix 1 (c) there is no strong contact between CA-NTD and CA-CTD (line 263-264). The studied EIAV assemblies are rather small: narrow tube 35nm, wide tube 70nm and spherical particles are 100nm. In general, retroviruses have a larger dimension. Is it possible that these missing contacts actually present in the authentic particles?

a) We realize that our original description of the intra-hexameric CA_NTD_ in EIAV was misleading. There are intra-hexameric CA_NTD_ interactions; however, they are not required to establish the hexamer. These interactions are highly variable (or even absent) among the tubular and spherical assemblies. To avoid any confusion, we have now rephrased this section in the manuscript as follows:

“*Taken together these results identify key interaction domains in the EIAV immature lattice. Although intra-hexameric CANTD interactions can be formed in EIAV, their high structural variability suggests that the CANTD is not required to establish intra-hexameric interactions, but rather forms a trimer to promote interactions with neighboring hexamers. Intra-hexameric interactions are maintained by the CACTD and the CA-SP 6HB*.”

The spherical EIAV assemblies, with a diameter of 100 nm, would form virus particles (which additionally have MA and membrane) with a diameter closer to 120 nm. This is comfortably within the range of diameters seen for retrovirus particles. We note also that HIV dMACANC VLPs , of ~100nm diameter, preserve the same set of interactions as purified immature viruses (Schur et al., 2016). For these reasons we believe the interactions formed in the spherical EIAV VLPs are very likely representative of the interactions in immature EIAV particles.

4) Line 109-110: The authors suggest that “the retroviral CA-NTD and CA-CTD are independent structural entities that act autonomously in regulating immature virus particle diameter and curvature”. This idea was repeated at line 363-364: “The spatial separation of EIAV CA-NTD and CA-CTD, and the lack of strong interactions between them, allow these two halves of CA to behave almost as independent entities, as suggested previously for other retroviruses”. This statement seems overly strong. In HIV-1, helix 11 and helix 8 interact with MHR and helix 7. This interaction would limit the orientation of NTD relative to CTD and the role of NTD in deciding the curvature of the Gag lattice.

In immature HIV-1 VLPs helix 11 and helix 8 do not interact with the MHR and helix 7. We expect that the reviewer is referring to the mature HIV-1 CA lattice where interactions between helix 8, helix 7, the MHR and helix 11 can be observed. We are therefore comfortable with our conclusion that no strong interactions between CA_NTD_ and CA_CTD_ are formed in the immature EIAV lattice.

5) Line 292-295: Line 292 lists the SP sequence as (LAKAL) in which the residue 359 is L. However in line 295, the last residue is listed as T395. Please clarify.

We thank the reviewer for spotting this mistake. It should read L359. We have corrected this in the revised version of the manuscript

6) Supplement figure 4, the electron density looks low resolution. A better figure is desired.

Supplemental figure 4A is filtered to 8 Å to allow easier observation of the individual domains (CA_NTD_/CA_CTD_/SP). Also, during submission of the manuscript the quality of this figure was reduced in the final PDF. We will ensure that this figure is reproduced with sufficient quality.

Additional changes to the manuscript:

We have in addition removed the following sentence (Line 136):

*“Increasing the length of the oligo did not alter assembly as determined by negative stain TEM analysis (data not shown).”*

This sentence was removed in accordance with the PLOS Pathogens Author Guidelines, as PLOS Pathogens does not publish references to “data not shown”.

The observation that oligo length does not alter assembly is a very technical point (not changing in any way the conclusions of our manuscript) and hence, in our opinion, does not warrant an additional figure. We therefore opted to remove this sentence and hope this is acceptable.
